# Supplementary material for: COVID-19 Vaccine Hesitancy among U.S. Veterans Experiencing Homelessness in Transitional Housing
Source: Int J Environ Res Public Health. 2022 Nov 29;19(23):15863. doi: 10.3390/ijerph192315863 (PMC9735876; doi:10.3390/ijerph192315863)
Supplement: Supplementary file 1 [file ijerph-19-15863-s001.zip › ijerph-2006278-supplementary.pdf]

Supplement S1: Interview Questionnaire Tool

BACKGROUND

We would like to start with a background question.

1. Can you tell me briefly about your experience in the GPD program? How long have you been in the program? What are you living arrangements? Dining arrangements?

COVID-19

We would now like to ask about Covid-19.

2. How worried are you about Covid-19? (Could include getting infected, getting sick, or dying)

3. On a typical day, are you ever in an environment where you might be exposed to COVID-19?
   a. Probe: How is this influenced by your work/school/family contexts

4. Can you tell me some of the things that make you (worried/not worried) about Covid-19?

5. How likely are you to get the Covid-19 vaccine when it becomes available to you?
On a scale of 1-5
   1= Definitely Not
   2= Probably Not
   3= Don't Know/Neutral/Don't have enough information
   4= Probably Yes
   5= Definitely Yes
   6= Is Already Vaccinated

6. What are your reasons for feeling this way?
   A) If you had a choice, would you be more willing to receive one brand versus another (e.g. Moderna, Pfizer, or Johnson & Johnson)?
   B) IF YES, possible prompts could be: Advice from health care provider; Protection against severe illness; Possible pre-existing conditions among self or others
   C) IF NO, possible prompts could be: Worry about the side effects; Too busy; Lack of trust in government; Don't like shots, discomfort)

7. [IF WILLING TO GET THE VACCINE] If you were to try to get the Covid-19 vaccine, do you anticipate having any difficulty going through with the process? For example:
   a. Prompts: Time, technology access (cell phone or internet for appt), availability, transportation, work schedule, family responsibilities)
   b. Does your willingness to get the vaccine depend on where you would get it?

INFORMATION SOURCES AND ATTITUDES

Now we would like to ask you some questions about sources of information about the COVID vaccine

8. Where have you received most of your information about the Covid-19 vaccine?
   a. prompt on TV, internet, radio, newspapers, friends/family
   b. what sources of information do you trust most?

9. Do you regularly receive care from a specific healthcare provider? (VA or other?)

10. Has your health care provider talked with you about the Covid-19 vaccine?
  a. Have they recommended that you get the Covid-19 vaccine?
  b. What kind of information did you receive from your health care provider; what did they tell you?
  c. Do you generally trust the information you get from your health care provider?
  d. Have you ever felt disrespected or mistreated by a health care provider (e.g. in regards to your appearance, your education, cultural background, etc.), enough so that you considered not returning to that health care facility?
  e. IF RESPONDENT INDICATED A NON-VA PROVIDER AS PRIMARY: Have you received information from the VA about the vaccine? Do you generally trust the information you receive from the VA?

11. Can you tell me about your experiences with your GPD program during the COVID-19 pandemic?
  a. How well do you feel your GPD has handled COVID-19 and handled safety issues during the pandemic?
  b. Are you concerned about contracting COVID-19 within your facility?

12. Have you gotten any information from your GPD about the vaccine?
  a. How do you feel about the trustworthiness of health care information you get from your GPD?

13. Have you had conversations with your fellow Veterans in the GPD about the vaccine? Do you have any sense about whether they are likely to get the vaccine when it becomes available?

14. [ASK IF DON'T WANT VACCINE] What else, if anything, might ease your worry and concerns about getting the vaccine?
  a. If you don't want the vaccine now or are not sure, what would have to change for you to decide to get the vaccine?
  b. Are there any sources of information that would make you more willing to get the vaccine?

General Vaccination Attitudes & Behaviors

Next, we would like to ask you a couple of questions about your feelings about vaccination.

15. Did you get a flu shot either during this current flu season or the year before?
  a. *IF YES: Where do you usually receive it?*

16. Could you tell us what your reasons for (receiving/not receiving) the flu vaccine in these past years?

17. Apart from the COVID-19 vaccine, how do you feel about vaccines in general? (Example: the flu shot)
  a. Have you ever been reluctant or hesitated to get a vaccination?
  b. Have you ever refused a vaccination? If yes, which vaccine(s) did you refuse?
  c. Do you believe that vaccines can protect from serious diseases?

SOCIODEMOGRAPHIC INFORMATION

If it's okay with you, we would like to ask a couple of questions about demographics.

18. Do you consider yourself to be in a medically high-risk category, due to your age or any health conditions you might have that may put you at risk of COVID?
  a. E.g. obesity, heart disease, diabetes, high blood pressure, asthma, lung disease (COPD), older adults

19. What is your age? Or feel free to give us an age range. Are you in your:

20s
30s
40s
50s
60s
70s

20. Which of the following best describes you?

Asian or Pacific Islander
Black or African American
Hispanic or Latino
Native American or Alaskan Native
White or Caucasian
Multiracial or Biracial
Race/ethnicity not mentioned

CLOSING

21. Is there anything else that we haven't asked you today, that you'd like to share with us, about your opinions of the Covid-19 vaccines?
